# Ethyl (2*Z*)-3-hy­droxy-3-(4-nitro­phen­yl)prop-2-enoate

**DOI:** 10.1107/S1600536814011891

**Published:** 2014-06-04

**Authors:** Tania N. Hill, Naadiya Patel

**Affiliations:** a Molecular Sciences Institute, School of Chemistry, University of the Witwatersrand, PO WITS 2050, Johannesburg, South Africa

## Abstract

The title compound, C_11_H_11_NO_5_, is essentially planar, with an r.m.s. deviation of 0.06 Å. The mol­ecular structure is stabilized by an intra­molecular O—H⋯O hydrogen bond. In the crystal, molecules are linked by two pairs of C—H⋯O hydrogen bonds, forming sheets, lying parallel to (101), which enclose *R*_4_^4^(26) ring motifs.

## Related literature   

For similar crystal structures, see: Caracelli *et al.* (2010 ▶); Yin *et al.* (2004 ▶); Syu *et al.* (2010 ▶). For geaph-set motifs, see: Bernstein *et al.* (1995 ▶).
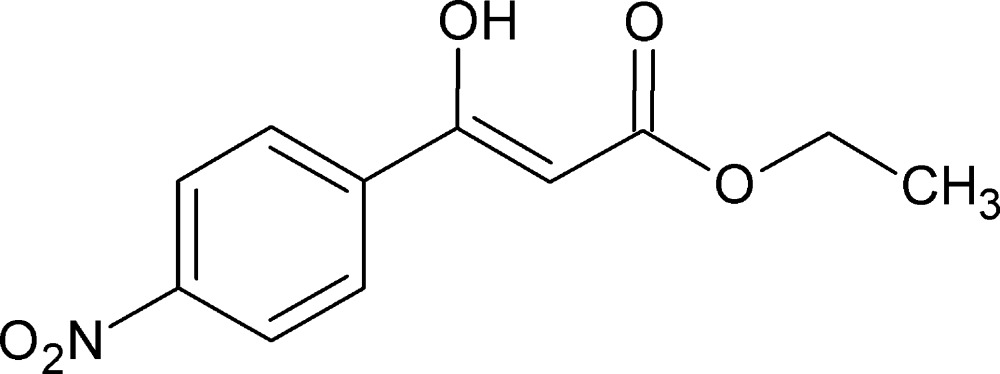


## Experimental   

### 

#### Crystal data  

C_11_H_11_NO_5_
*M* *_r_* = 237.21
Monoclinic, 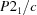

*a* = 13.0495 (9) Å
*b* = 10.8363 (6) Å
*c* = 7.6723 (5) Å
β = 91.268 (4)°
*V* = 1084.66 (12) Å^3^
*Z* = 4
Mo *K*α radiation
μ = 0.12 mm^−1^
*T* = 173 K
0.34 × 0.21 × 0.17 mm

#### Data collection  

Bruker APEXII CCD area-detector diffractometer
Absorption correction: multi-scan (*SADABS*; Bruker, 2004 ▶) *T*_min_ = 0.962, *T*_max_ = 0.981
10438 measured reflections
2620 independent reflections
1476 reflections with *I* > 2σ(*I*)
*R*_int_ = 0.057

#### Refinement  

*R*[*F* ^2^ > 2σ(*F* ^2^)] = 0.047
*wR*(*F* ^2^) = 0.129
*S* = 1.03
2620 reflections
156 parameters
H-atom parameters constrained
Δρ_max_ = 0.21 e Å^−3^
Δρ_min_ = −0.15 e Å^−3^

### 

Data collection: *APEX2* (Bruker, 2005 ▶); cell refinement: *SAINT-Plus* (Bruker, 2004 ▶); data reduction: *SAINT-Plus* and *XPREP* (Bruker, 2004 ▶); program(s) used to solve structure: *SHELXS97* (Sheldrick, 2008 ▶); program(s) used to refine structure: *SHELXL97* (Sheldrick, 2008 ▶); molecular graphics: *DIAMOND* (Brandenburg & Putz, 2005) ▶; software used to prepare material for publication: *WinGX* (Farrugia, 2012 ▶).

## Supplementary Material

Crystal structure: contains datablock(s) global, I. DOI: 10.1107/S1600536814011891/bx2458sup1.cif

Structure factors: contains datablock(s) I. DOI: 10.1107/S1600536814011891/bx2458Isup2.hkl

Supporting information file. DOI: 10.1107/S1600536814011891/bx2458Isup3.cml

CCDC reference: 1004533

Additional supporting information:  crystallographic information; 3D view; checkCIF report

## Figures and Tables

**Table 1:** Hydrogen-bond geometry (Å, °)

*D*—H⋯*A*	*D*—H	H⋯*A*	*D*⋯*A*	*D*—H⋯*A*
O1—H1⋯O2	0.84	1.87	2.6028 (16)	146
C2—H2⋯O5^i^	0.95	2.5	3.362 (2)	150
C8—H8⋯O2^ii^	0.95	2.57	3.5050 (17)	170

Symmetry codes: (i) 
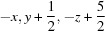
; (ii) 
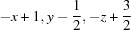
.

**Fig. 1.**
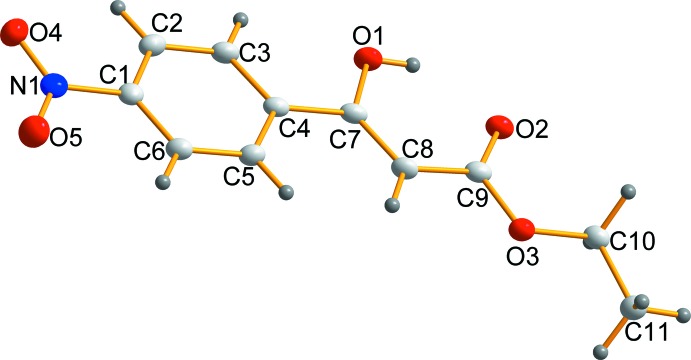
Molecular structure of (I), showing the atom labelling scheme and displacement ellipsoids at 20% probability level. (arbitrary spheres for the H atoms)

**Fig. 2.**
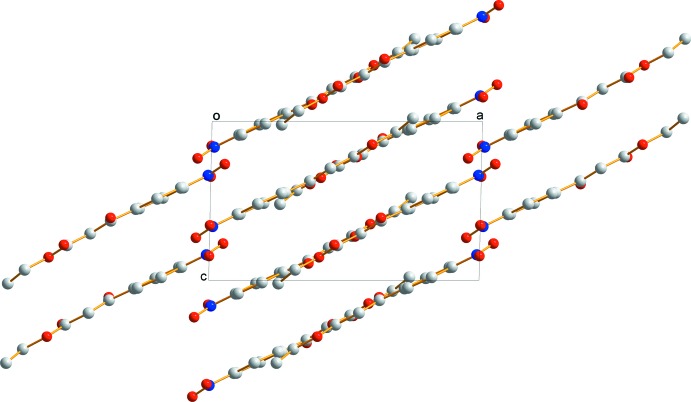
Packing of (I) viewed along the b-axis. Hydrogen atoms omitted for clarity.

## supplementary crystallographic information

### S1. Comment

The molecular structure of (I) is illustrated in Figure 1, and was obtained by recrystallization of the commercially available compound. The title compound, C_11_H_11_NO_5_, consists of a hydroxy (O1) and a *p*-nitrophenyl substituted propenoate.The molecule is essentially planar with an *r.m.s.* deviation of 0.065Å, the larger *r.m.s.* value is as a result of the slight twisting of the substituents on the propenoate backbone, the dihedral angle of the planes of the subsitutents with the propenoate plane were found to be 3.69 (4) ° for the *p*-nitrophenyl and 3.3 (1) ° for the ethyl ester.

The propenoate backbone was observed in the enol tautomeric form with a typical hydrogen bond interaction between the hydroxy (O1) and the carbonyl (O2) with a distance of 2.603 (2) Å. The packing of (I) is seen as parallel sheets (Figure 2) when viewed along the *b*-axis. The crystal and molecular structure is stabilized by two weak C—H···O hydrogen bond interactions with graph-set motif R_4_^4^(26) (Bernstein, *et al.*, 1995) and one O—H···O intramolecular hydrogen bond interaction respectively, Table 1

### S2. Experimental

Ethyl 4-nitrobenzoylacetate was obtained commercially. (I) It was redissolved in warm MeOH and allowed to cool to room terperature. Yellow crystals suitable for single-crystal diffraction were obtained by slow evaporation over a few days.

### S3. Refinement

All hydrogen atoms were positioned geometrically and refined using a riding model, with C—H = 0.95 Å *U*_iso_(H)= 1.2 *U*_eq_(C) for the aromatic H atoms, with C—H = 0.98 Å *U*_iso_(H)= 1.5 *U*_eq_(C) for methyl H atoms and with O—H = 0.84 Å *U*_iso_(H)= 1.5 *U*_eq_(O) for the hydroxyl H atoms. The methyl and hydroxyl groups were allowed to rotate with a fixed angle arround the C—C bond to best fit the experimental electron density [HFIX 137 and HFIX 147 in *SHELXL97* (Sheldrick, 2008)].

### Crystal data

**Table:**

C_11_H_11_NO_5_	*F*(000) = 496
*M**_r_* = 237.21	*D*_x_ = 1.453 Mg m^−^^3^
Monoclinic, *P*2_1_/*c*	Mo *K*α radiation, λ = 0.71073 Å
Hall symbol: -P 2ybc	Cell parameters from 2434 reflections
*a* = 13.0495 (9) Å	θ = 2.4–24.9°
*b* = 10.8363 (6) Å	µ = 0.12 mm^−^^1^
*c* = 7.6723 (5) Å	*T* = 173 K
β = 91.268 (4)°	Cuboid, yellow
*V* = 1084.66 (12) Å^3^	0.34 × 0.21 × 0.17 mm
*Z* = 4	

### Data collection

**Table:**

Bruker APEXII CCD area-detector diffractometer	2620 independent reflections
Radiation source: fine-focus sealed tube	1476 reflections with *I* > 2σ(*I*)
Graphite monochromator	*R*_int_ = 0.057
Detector resolution: 512 pixels mm^-1^	θ_max_ = 28°, θ_min_ = 1.6°
ω scans	*h* = −17→14
Absorption correction: multi-scan (*SADABS*; Bruker, 2004)	*k* = −14→12
*T*_min_ = 0.962, *T*_max_ = 0.981	*l* = −6→10
10438 measured reflections	

### Refinement

**Table:**

Refinement on *F*^2^	0 restraints
Least-squares matrix: full	H-atom parameters constrained
*R*[*F*^2^ > 2σ(*F*^2^)] = 0.047	*w* = 1/[σ^2^(*F*_o_^2^) + (0.0505*P*)^2^ + 0.0768*P*] where *P* = (*F*_o_^2^ + 2*F*_c_^2^)/3
*wR*(*F*^2^) = 0.129	(Δ/σ)_max_ < 0.001
*S* = 1.03	Δρ_max_ = 0.21 e Å^−^^3^
2620 reflections	Δρ_min_ = −0.15 e Å^−^^3^
156 parameters	

### Special details

**Table:**

Geometry. All e.s.d.'s (except the e.s.d. in the dihedral angle between two l.s. planes) are estimated using the full covariance matrix. The cell e.s.d.'s are taken into account individually in the estimation of e.s.d.'s in distances, angles and torsion angles; correlations between e.s.d.'s in cell parameters are only used when they are defined by crystal symmetry. An approximate (isotropic) treatment of cell e.s.d.'s is used for estimating e.s.d.'s involving l.s. planes.

### Fractional atomic coordinates and isotropic or equivalent isotropic displacement parameters (Å^2^)

**Table:**

	*x*	*y*	*z*	*U*_iso_*/*U*_eq_	
C1	0.10142 (14)	0.26271 (15)	1.0840 (2)	0.0434 (4)	
C2	0.11032 (14)	0.38942 (15)	1.0826 (2)	0.0488 (5)	
H2	0.0579	0.4402	1.128	0.059*	
C3	0.19712 (15)	0.44047 (15)	1.0135 (2)	0.0466 (5)	
H3	0.2041	0.5277	1.0101	0.056*	
C4	0.27481 (13)	0.36667 (13)	0.9487 (2)	0.0393 (4)	
C5	0.26256 (14)	0.23883 (14)	0.9526 (2)	0.0430 (4)	
H5	0.3148	0.1872	0.9085	0.052*	
C6	0.17583 (14)	0.18653 (14)	1.0193 (2)	0.0446 (4)	
H6	0.1675	0.0994	1.0207	0.054*	
C7	0.36660 (14)	0.42403 (13)	0.87532 (19)	0.0406 (4)	
C8	0.44132 (14)	0.36221 (13)	0.7946 (2)	0.0427 (4)	
H8	0.4378	0.2748	0.7861	0.051*	
C9	0.52669 (14)	0.42678 (14)	0.7207 (2)	0.0419 (4)	
C10	0.68104 (14)	0.41084 (14)	0.5666 (2)	0.0454 (4)	
H10A	0.7235	0.4531	0.6569	0.055*	
H10B	0.6575	0.4729	0.4799	0.055*	
C11	0.74223 (15)	0.31324 (14)	0.4798 (2)	0.0517 (5)	
H11A	0.7682	0.2546	0.5675	0.078*	
H11B	0.8	0.3513	0.4206	0.078*	
H11C	0.6987	0.2696	0.3942	0.078*	
N1	0.01035 (12)	0.20690 (15)	1.16117 (19)	0.0539 (4)	
O1	0.36768 (10)	0.54740 (9)	0.89365 (15)	0.0503 (4)	
H1	0.421	0.5761	0.8495	0.075*	
O2	0.53793 (9)	0.54001 (9)	0.72351 (14)	0.0481 (4)	
O3	0.59352 (9)	0.35202 (9)	0.64570 (14)	0.0445 (3)	
O4	−0.05048 (11)	0.27473 (14)	1.2318 (2)	0.0750 (5)	
O5	0.00065 (11)	0.09522 (13)	1.15338 (18)	0.0731 (4)	

### Atomic displacement parameters (Å^2^)

**Table:**

	*U*^11^	*U*^22^	*U*^33^	*U*^12^	*U*^13^	*U*^23^
C1	0.0507 (12)	0.0458 (10)	0.0334 (9)	−0.0012 (8)	−0.0024 (8)	−0.0002 (7)
C2	0.0536 (13)	0.0475 (10)	0.0451 (10)	0.0114 (9)	−0.0014 (9)	−0.0066 (8)
C3	0.0611 (13)	0.0334 (8)	0.0450 (10)	0.0044 (8)	−0.0037 (9)	−0.0036 (7)
C4	0.0516 (11)	0.0328 (8)	0.0334 (9)	0.0025 (7)	−0.0058 (8)	−0.0022 (6)
C5	0.0543 (12)	0.0355 (8)	0.0392 (10)	0.0044 (8)	0.0025 (8)	−0.0013 (7)
C6	0.0579 (12)	0.0355 (9)	0.0405 (10)	−0.0007 (8)	0.0006 (8)	−0.0012 (7)
C7	0.0579 (12)	0.0276 (8)	0.0358 (9)	0.0026 (7)	−0.0076 (8)	0.0003 (6)
C8	0.0572 (12)	0.0278 (8)	0.0430 (10)	−0.0013 (8)	−0.0022 (8)	0.0002 (7)
C9	0.0558 (12)	0.0342 (9)	0.0354 (10)	0.0021 (8)	−0.0065 (8)	0.0001 (7)
C10	0.0561 (12)	0.0384 (9)	0.0417 (10)	−0.0058 (8)	−0.0016 (8)	0.0028 (7)
C11	0.0601 (13)	0.0435 (9)	0.0519 (11)	−0.0023 (8)	0.0072 (9)	−0.0013 (8)
N1	0.0602 (12)	0.0604 (10)	0.0410 (9)	−0.0031 (8)	−0.0004 (8)	−0.0019 (7)
O1	0.0658 (10)	0.0291 (6)	0.0562 (8)	−0.0011 (5)	0.0053 (6)	−0.0010 (5)
O2	0.0628 (9)	0.0305 (6)	0.0510 (8)	−0.0022 (5)	0.0002 (6)	0.0005 (5)
O3	0.0546 (8)	0.0319 (6)	0.0471 (7)	−0.0002 (5)	0.0036 (6)	0.0002 (5)
O4	0.0628 (10)	0.0830 (10)	0.0799 (11)	0.0008 (8)	0.0175 (8)	−0.0198 (8)
O5	0.0831 (11)	0.0572 (9)	0.0796 (10)	−0.0103 (8)	0.0190 (8)	0.0101 (7)

### Geometric parameters (Å, º)

**Table:**

C1—C6	1.376 (2)	C8—C9	1.442 (2)
C1—C2	1.378 (2)	C8—H8	0.95
C1—N1	1.469 (2)	C9—O2	1.2358 (17)
C2—C3	1.377 (2)	C9—O3	1.3304 (19)
C2—H2	0.95	C10—O3	1.4526 (19)
C3—C4	1.392 (2)	C10—C11	1.491 (2)
C3—H3	0.95	C10—H10A	0.99
C4—C5	1.395 (2)	C10—H10B	0.99
C4—C7	1.472 (2)	C11—H11A	0.98
C5—C6	1.375 (2)	C11—H11B	0.98
C5—H5	0.95	C11—H11C	0.98
C6—H6	0.95	N1—O4	1.2179 (18)
C7—O1	1.3443 (17)	N1—O5	1.2181 (18)
C7—C8	1.345 (2)	O1—H1	0.84
			
C6—C1—C2	122.32 (16)	C7—C8—H8	119.6
C6—C1—N1	118.82 (15)	C9—C8—H8	119.6
C2—C1—N1	118.84 (16)	O2—C9—O3	122.24 (16)
C3—C2—C1	118.27 (16)	O2—C9—C8	124.55 (16)
C3—C2—H2	120.9	O3—C9—C8	113.20 (13)
C1—C2—H2	120.9	O3—C10—C11	108.01 (12)
C2—C3—C4	121.23 (15)	O3—C10—H10A	110.1
C2—C3—H3	119.4	C11—C10—H10A	110.1
C4—C3—H3	119.4	O3—C10—H10B	110.1
C3—C4—C5	118.57 (16)	C11—C10—H10B	110.1
C3—C4—C7	119.95 (14)	H10A—C10—H10B	108.4
C5—C4—C7	121.47 (15)	C10—C11—H11A	109.5
C6—C5—C4	120.87 (15)	C10—C11—H11B	109.5
C6—C5—H5	119.6	H11A—C11—H11B	109.5
C4—C5—H5	119.6	C10—C11—H11C	109.5
C5—C6—C1	118.72 (15)	H11A—C11—H11C	109.5
C5—C6—H6	120.6	H11B—C11—H11C	109.5
C1—C6—H6	120.6	O4—N1—O5	123.60 (17)
O1—C7—C8	122.50 (15)	O4—N1—C1	118.15 (15)
O1—C7—C4	112.74 (14)	O5—N1—C1	118.24 (16)
C8—C7—C4	124.74 (14)	C7—O1—H1	109.5
C7—C8—C9	120.89 (14)	C9—O3—C10	116.26 (12)
			
C6—C1—C2—C3	0.0 (2)	C5—C4—C7—C8	5.6 (2)
N1—C1—C2—C3	178.54 (14)	O1—C7—C8—C9	−0.9 (2)
C1—C2—C3—C4	−0.8 (2)	C4—C7—C8—C9	177.59 (14)
C2—C3—C4—C5	0.9 (2)	C7—C8—C9—O2	−0.6 (2)
C2—C3—C4—C7	179.86 (14)	C7—C8—C9—O3	−179.97 (14)
C3—C4—C5—C6	−0.1 (2)	C6—C1—N1—O4	173.58 (15)
C7—C4—C5—C6	−179.12 (14)	C2—C1—N1—O4	−5.0 (2)
C4—C5—C6—C1	−0.6 (2)	C6—C1—N1—O5	−5.3 (2)
C2—C1—C6—C5	0.7 (2)	C2—C1—N1—O5	176.14 (15)
N1—C1—C6—C5	−177.83 (14)	O2—C9—O3—C10	−0.1 (2)
C3—C4—C7—O1	5.2 (2)	C8—C9—O3—C10	179.31 (12)
C5—C4—C7—O1	−175.83 (14)	C11—C10—O3—C9	−176.13 (13)
C3—C4—C7—C8	−173.38 (15)		

### Hydrogen-bond geometry (Å, º)

**Table:**

*D*—H···*A*	*D*—H	H···*A*	*D*···*A*	*D*—H···*A*
O1—H1···O2	0.84	1.87	2.6028 (16)	146
C2—H2···O5^i^	0.95	2.5	3.362 (2)	150
C8—H8···O2^ii^	0.95	2.57	3.5050 (17)	170

Symmetry codes: (i) −*x*, *y*+1/2, −*z*+5/2; (ii) −*x*+1, *y*−1/2, −*z*+3/2.
